# Zebrafish toxicological screening could aid Leishmaniosis drug discovery

**DOI:** 10.1186/s42826-021-00104-1

**Published:** 2021-09-16

**Authors:** Hirla Costa Silva Fukushima, Ricardo Lacava Bailone, Tatiana Corrêa, Helena Janke, Luís Kluwe De Aguiar, Princia Grejo Setti, Ricardo Carneiro Borra

**Affiliations:** 1 Center of Biological and Health Sciences, Federal University of Sao Carlos, Washington Luis Road km 235, Sao Carlos, 13565-905 Brazil; 2 Department of Federal Inspection Service, Ministry of Agriculture, Livestock and Supply of Brazil, Federal Inspection Service,, Treze de Maio, Street n°1558, Bela Vista, São Paulo 01327-00 Brazil; 3 Department of Genetic and Evolution, Federal University of São Carlos, Washington Luis Road km 235, São Carlos, 13565-905 Brazil; 4 Department of Food Technology and Innovation, Harper Adams University, Edgmond, Newport, TF10 8NB UK

**Keywords:** 3Rs, Animal health, Human health, Immunity, Leishmaniose, Toxicology

## Abstract

**Background:**

Recently a screen from a library of 1.8 million compounds identified in vitro a potent activity of the 2-aminobenzimidazoles series against *Leishmania infantum,* the etiological agent responsible by over 20.000 deaths each year*.* Several analogs were synthesized and in vitro tested through an optimization program, leading to a promising 2-aminobenzimidazoles derived compound (2amnbzl-d) that was progressed to in vivo mice studies. However, the not expected toxic effects prevented its progression to more advanced preclinical and clinical phases of drug development. Due to limitations of cell models in detecting whole organism complex interactions, 90% of the compounds submitted to pre-clinical tests are reproved. The use of Zebrafish embryo models could improve this rate, saving mammals, time and costs in the development of new drugs. To test this hypothesis, we compared 2amnbzl-d with two compounds with already established safety profile: carbamazepine and benznidazole, using an embryo Zebrafish platform based on acute toxicity, hepatotoxicity, neurotoxicity and cardiotoxicity assays (Pltf-AcHpNrCd).

**Results:**

Tests were performed blindly, and the results demonstrated the presence of lethal and teratogenic effects (CL50%: 14.8 µM; EC50%: 8.6 µM), hepatotoxic in concentrations above 7.5 µM and neurotoxic in embryos exposed to 15 µM of 2amnbzl-d. Nevertheless, benznidazole exposition showed no toxicity and only the 100 µM of carbamazepine induced a bradycardia.

**Conclusions:**

Results using Pltf-AcHpNrCd with zebrafish reproduced that found in the toxicological tests with mammals to a portion of the costs and time of experimentation.

## Background

Currently, the tests used in toxicological screening are costly, time-consuming, and not satisfactorily predictive, thus toxicity is one of the major attritions causes during the drug development process. The placement of a new drug on the market takes about 8 years, with a cost of approximately US $ 1 billion [1]. Despite this, about 90% of the drugs in development fail in the clinical stages and are discontinued, leading to the loss of a large volume of investment [2]. Considering this problem, there is a recurring discussion about improving the risk prediction of new compound in the early stages of its development to optimize the development of new drugs and the use of animals in regulatory trials.

In vitro studies with cell cultures are poor predictors of drug absorption, distribution, metabolism and excretion (ADME) in whole organism, on the other hand, in vivo studies help to understand the possible toxic properties of new drugs, but they are time-consuming, expensive and use a limited number of animals. Zebrafish embryos and larvae with up to 120 h of life have been considered unprecedented toxicological models, which exhibit a diverse repertoire of biological processes and possess fully integrated vertebrate organ systems [3]. Thus, zebrafish can bridge the gap between in vitro safety assays and mammals’ models in a fast and cost-effective manner and would have a key role in accelerating the process of new chemicals development, improving prediction, prioritizing safe compounds, and decreasing testing time and costs substantially [4–6].

This model has the advantage of having OECD-specific guidelines for safety evaluation of chemical compounds (acute toxicity), which is performed within 96 h [7]. It’s also an excellent model organism to study liver damage induced by chemical compounds due to the high degree of genetic conservation for enzymes and pathways necessary in drug metabolism, such as ARH receptors, CYP enzymes or ADH isoenzymes, present and functional since the early stages of development [5, 8]. In the same way, cardiovascular physiology is also highly conserved between humans and zebrafish at anatomical, cellular, and membrane-biology levels, thus many human cardiovascular drugs have been shown to have identical effects on larvae zebrafish physiology, and numerous human cardiovascular disorders have been recapitulated in zebrafish genetic model [3, 4]. The neural activity can be assessed by embryo neurobehavioral biomarkers, related to the neuromuscular function of the central nervous system that can indicate possible changes in neural development, such as synaptogenesis and glial cell growth [9]. Thus, the three main reasons behind the retirement of drugs in clinical phases and post market withdrawal could possibly have been predicted in embryo and larvae zebrafish trials in the early stages of development and saved mammals models (mice, rabbits, dogs, monkeys) and billions of dollars invested.

Ferreira et al. [10] recently published the results of a hit-to-lead campaign aiming at the identification of new preclinical candidates for the treatment of leishmaniasis, a potentially fatal neglected disease that affects millions of people worldwide and whose current treatment options are limited either by length of treatment or toxic side effects. Starting from a promising 2-aminobendazole hit identified via an in vitro phenotypic screen, hundreds of analogues were synthetized with the aim of finding a molecule capable of killing the parasite and be human safe. From these, two promising leads showing a good balance of properties were progressed to a 5-day tolerability study in healthy mice. The 25 mg/kg/day dose was well tolerated, but animals treated with 50 mg/kg/day or above showed clinical signs of toxicity. During the follow up study in mice infected with Leishmania, results showed lack of efficacy for both compounds and a poor safety profile for the 2amnbzl-d lead.

The aim of this study was to evaluate whether the toxicity identified during the in vivo studies with the new chemical entity 2amnbzl-d published by Ferreira et al. [10] could be predicted by tests using the zebrafish model and reduced the time and cost with the tests, as well as the use of mammals in research of drug discovery, thus impacting strongly in the 3Rs principles (replacement, reduction and refinement).

## Results

**Fig. 1:**
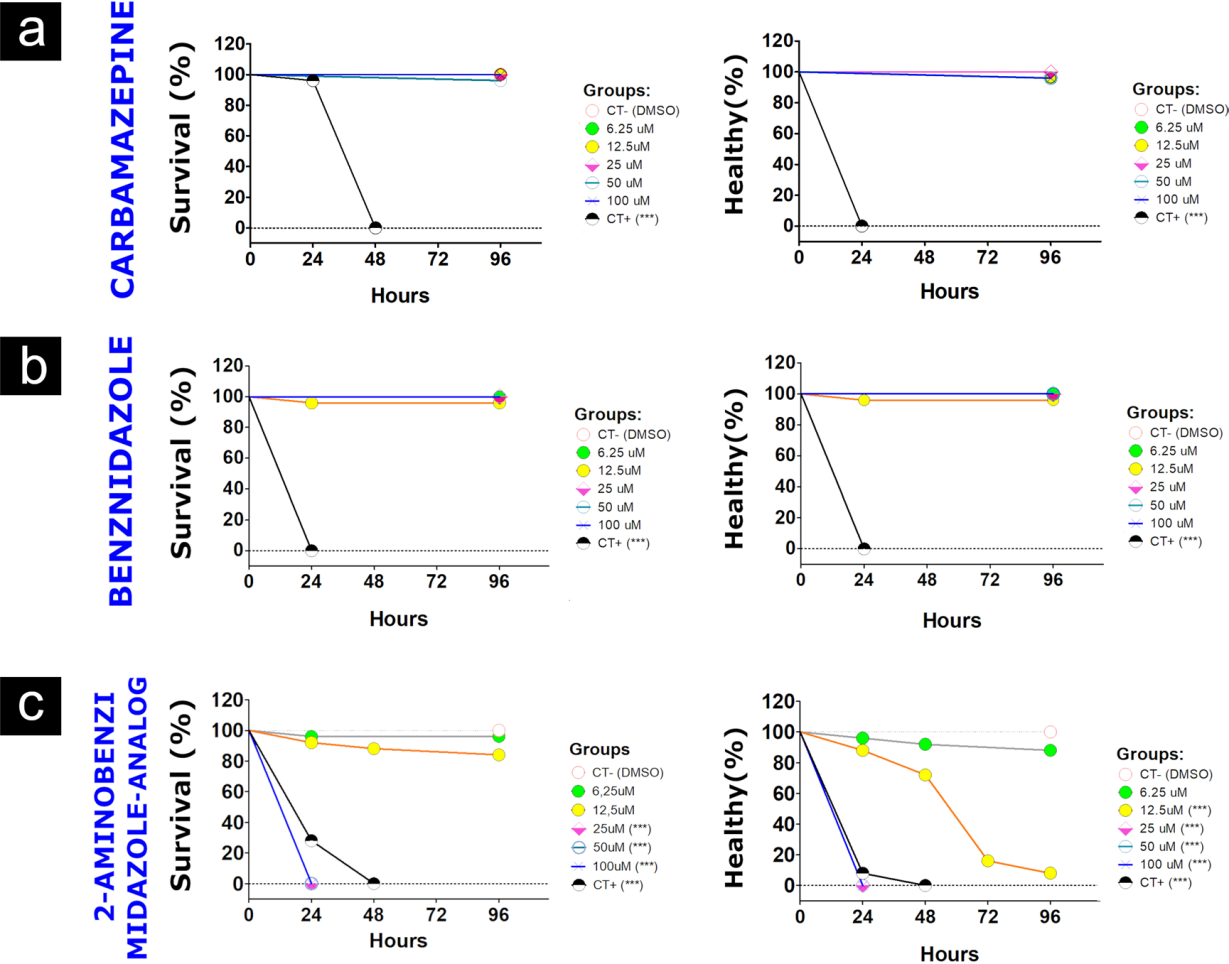
Survival graph relating the percentage of survival of embryos and health embryos exposed to their respective concentrations of carbamazepine (**a**), benznidazole (**b**) and 2-aminobenzimidazole-derived (**c**) by 96 hpf, compared to the DMSO control. To analyze the results, Log-rank Statistical Test (Mantel-Cox) was performed to compare all groups with the DMSO control. The *p* values were corrected by the Bonferroni methodology and considered significant for values *p* < 0.001

**Fig. 2:**
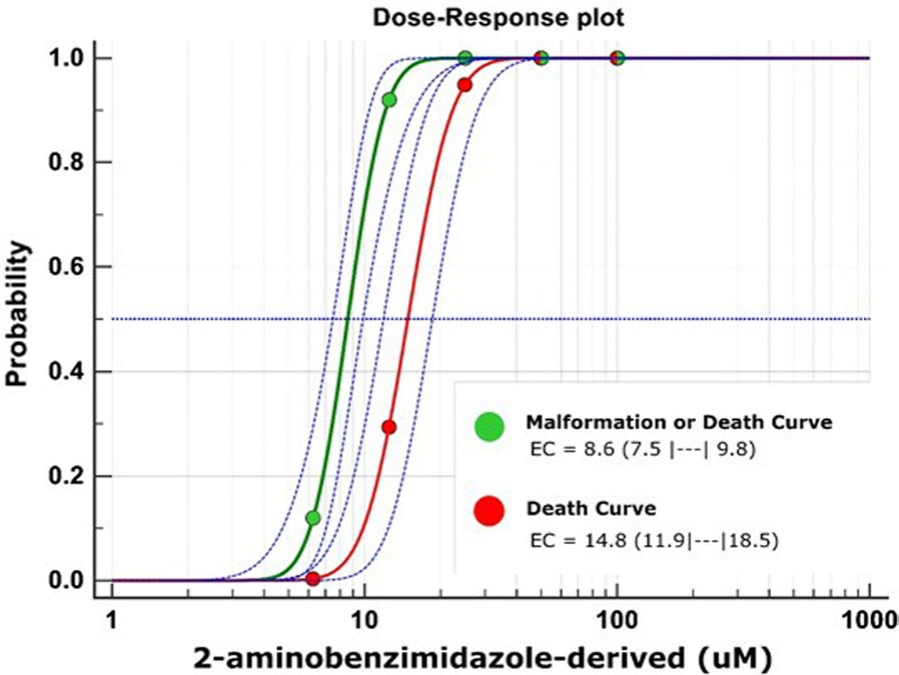
Dose–response curves showing the percentages of death or malformation embryos after treatment with each concentration (6.25, 12.5, 25, 50, 100 µM) of 2-aminobenzimidazole-derived for 96 h. The red line represented the fit model calculated by Probit Regression (R^2^ = 0.865, *p* < 0.0001) of death embryos and its confidence interval of 95% (IC95) (dashed blue line). Horizontal line represented the EC50 value of 14.8 µM. The green line represented the fit model calculated by Probit Regression (R^2^ = 0.855, *p* < 0.0001) of death or malformation embryos and its IC95 (dashed blue line) calculated by Medcalc Software. Horizontal line represented the EC50 value of 8.6 µM

The acute toxicity of the compounds, performed using the OECD guideline 236, demonstrated that embryos exposed to 2amnbzl-d in concentrations above 25 µM presented lethality significantly different from the DMSO control group. The cumulative mortality for each concentration can be seen in Fig. 1. The LC50% for 96 h found was 14.8 µM (Fig. 2). There was no mortality in the control groups, and there were no incidents during the tests that may have influenced the results. The tests carried out with exposure of embryos to carbamazepine and benznidazole compounds did not present acute toxicity in any tested concentration.

Teratogenic effects, represented by curve of malformations or death, can be seen in Fig. 1. At concentrations of 6.25 and 12.5 µM, the embryos remained alive for 96 h of evaluation, but most embryos exposed to 12.5 µM did not hatch, presented yolk sac hemorrhages and bradycardia (Fig. 4), which EC50% 8.57 µM. On the other hand, carbamazepine and benznidazole did not demonstrate a teratogenic effect on zebrafish embryos development at any concentration tested.

**Fig. 3:**
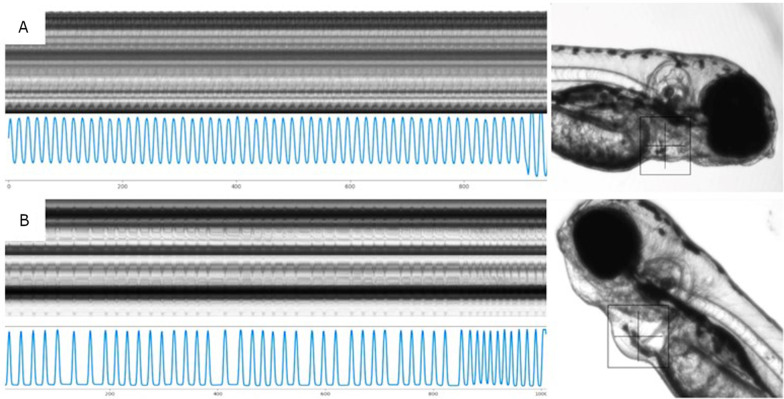
Example of cardiographs, representing the time series of patterns of intensity (green channel from RGB) of the horizontal and vertical lines (1000 digitized frames), positioned on center of the heart´s larvae. Based on the image pattern formed, the rate and the rhythm of the heartbeat were calculated. In image (**A**), the constant rhythm of the heartbeat of a larva exposed to 12.5 µM of 2-aminobenzimidazole-derived can be observed, and in image (**B**) an altered rhythm of the positive control heartbeat of an exposed larva at 10.0 µM haloperidol (beat heating rate = 150 bpm, sample entropy = 1.05 and Poincaré: S = 3267) is showed. In this case, it is possible to observe variable time intervals of cardiac contractions (beat heating rate = 186 bpm, sample entropy = 0.11 and Poincaré: S = 8.28)

**Fig. 4:**
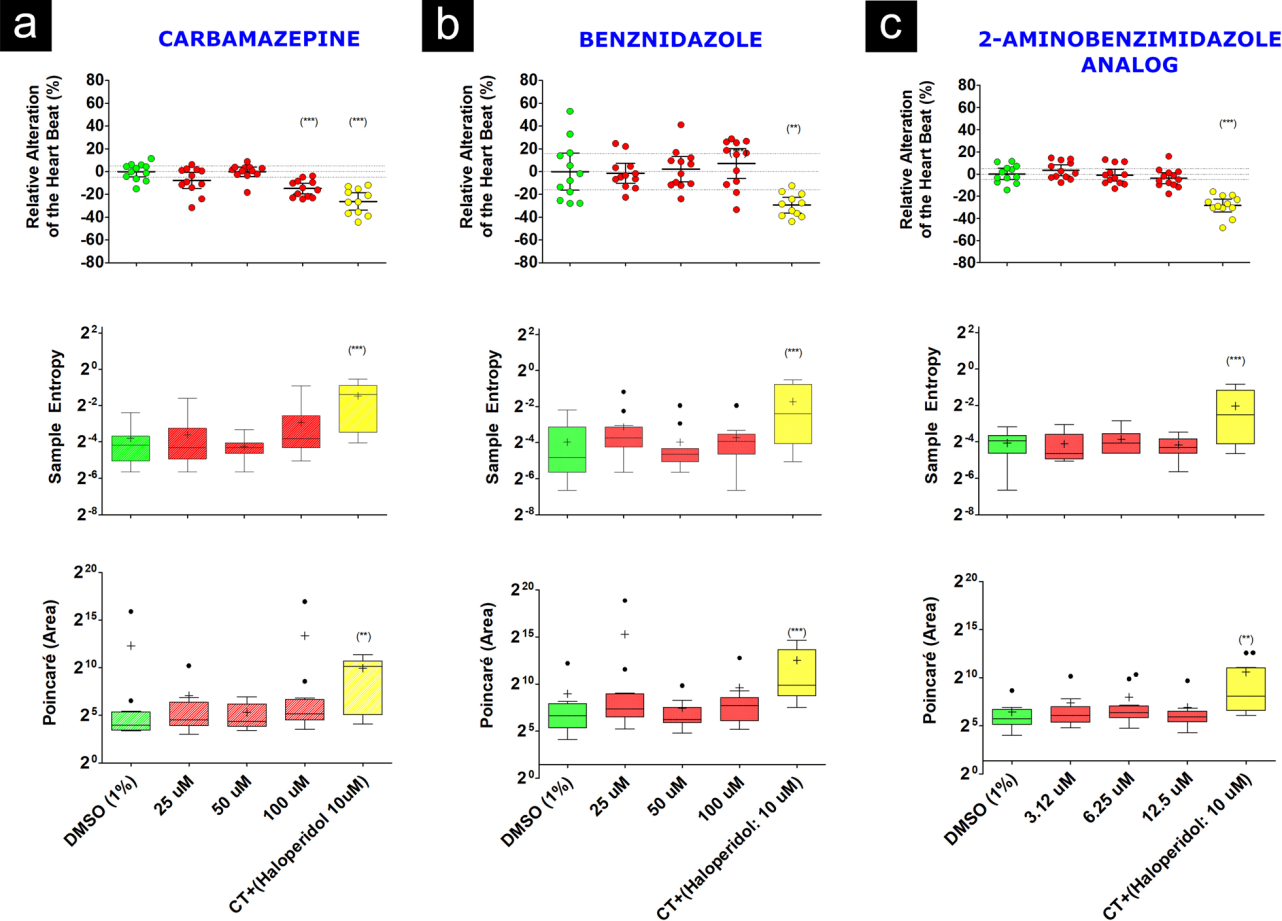
Scatter-plot showing the mean and distribution of the relative alteration of heart rate, and Tukey´s whiskers-plots showing the distribution of the sample entropy and Poincaré area values of *Danio rerio* larvae (96 hpf) exposed to different concentrations of the molecule carbamazepine (**a**), benznidazole (**b**) and from 2-aminobenzimidazole-derived (**c**). The data were analyzed using ANOVA statistical tests and Dunnett's Multiple Comparison Test. (***) *p* < 0.0001

To assess cardiotoxicity, hepatotoxicity and neurotoxicity, the maximum concentration tested of compound had as reference the LC50% previously obtained in the OECD 236 test. The heart rate data did not show differences between the control groups 1% DMSO and 2amnbzl-d groups or benznidazole groups. Zebrafish larvae exposed to 100 µM of carbamazepine showed bradycardia (Figs. 3 and 4).

**Fig. 5:**
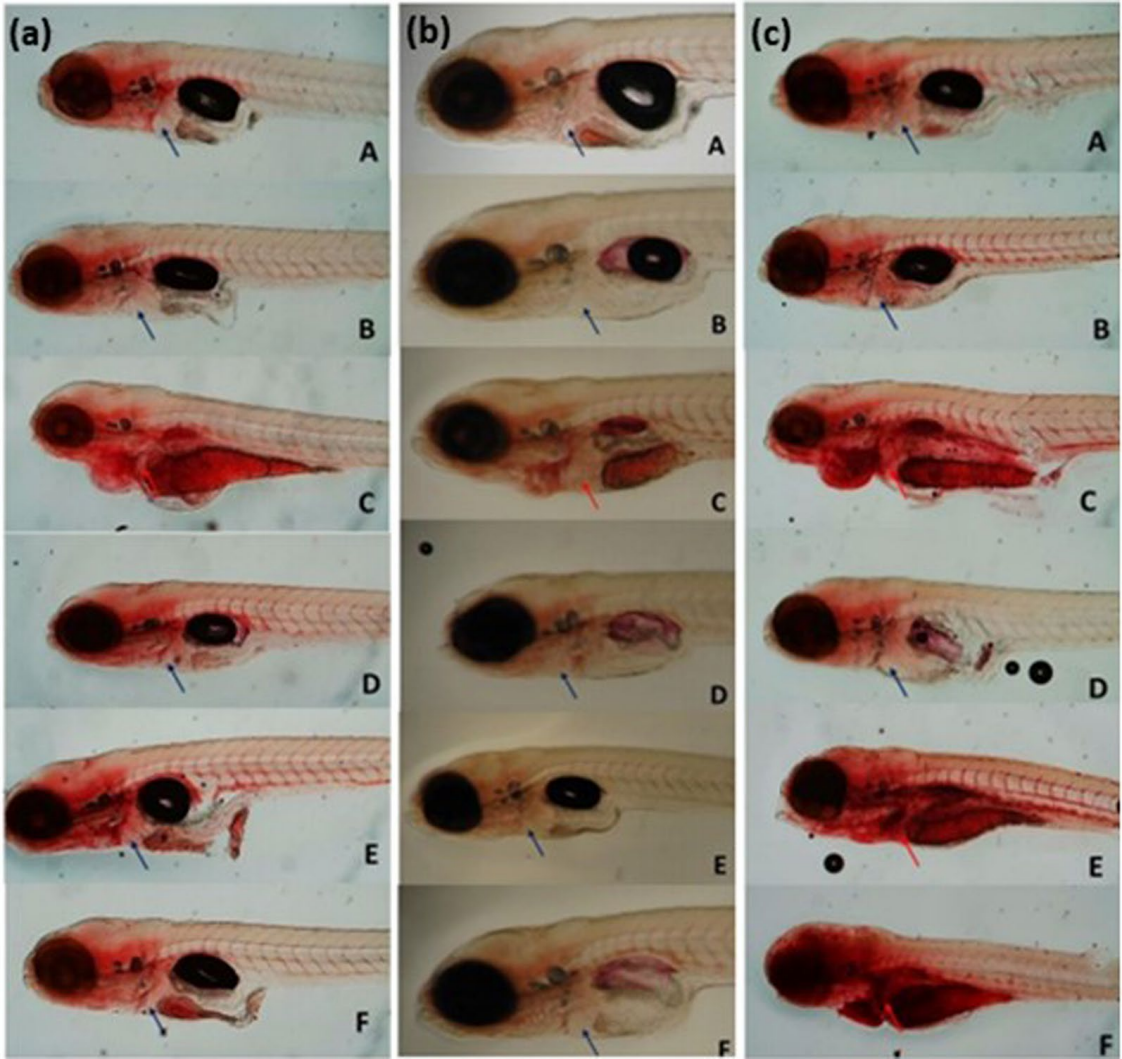
*Danio rerio* larvae 120 hpf stained with Oil Red O. Larvae exposed to the molecule carbamazepine (**a**), benznidazole (**b**) and 2-aminobenzimidazole derived (**c**) (72–120 hpf) (A) Negative control Medium ABNT [11]; (B) DMSO Control 1%, (C) Positive Control Ethanol 2% (D) Lower concentration; (E) Intermediate concentration; (F) Higher concentration. Blue arrows indicate the location of the unstained liver. Red arrows indicate the stained liver, with indication of hepatic alterations compatibles with steatosis

**Fig. 6:**
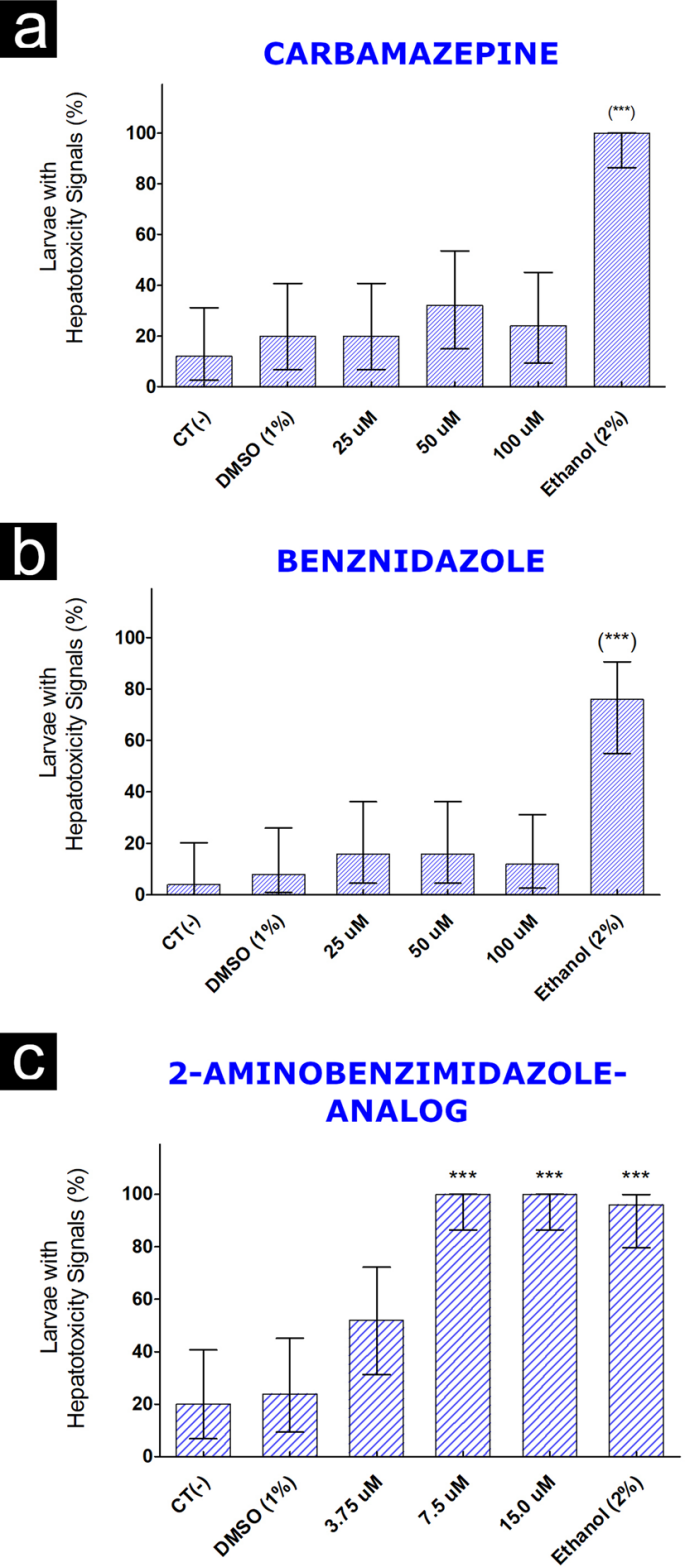
Percentage of *Danio rerio* Larvae 120 hpf (n = 25) exposed to carbamazepine (**a**), benznidazole (**b**) and 2-aminobenzimidazole derived (**c**) positive for hepatotoxicity (Statistical Proportion Test "exact" Clopper-Pearson confidence interval for the observed proportion Clopper & Pearson [12]; Fleis et al. [13] (***) *p* < 0.001 in comparison with DMSO (1%)

Regarding the hepatotoxic effects, no change in liver function was observed in groups of larvae exposed to the compound’s carbamazepine and benznidazole at all concentrations tested. On the other hand, the presence of red liver color and the increase in its size and/ or retention of the yolk sac, indicative of function liver changes, was verified in in larvae groups exposed to 2amnbzl-d concentrations above 7.5 µM (Figs. 5, 6).

**Fig. 7:**
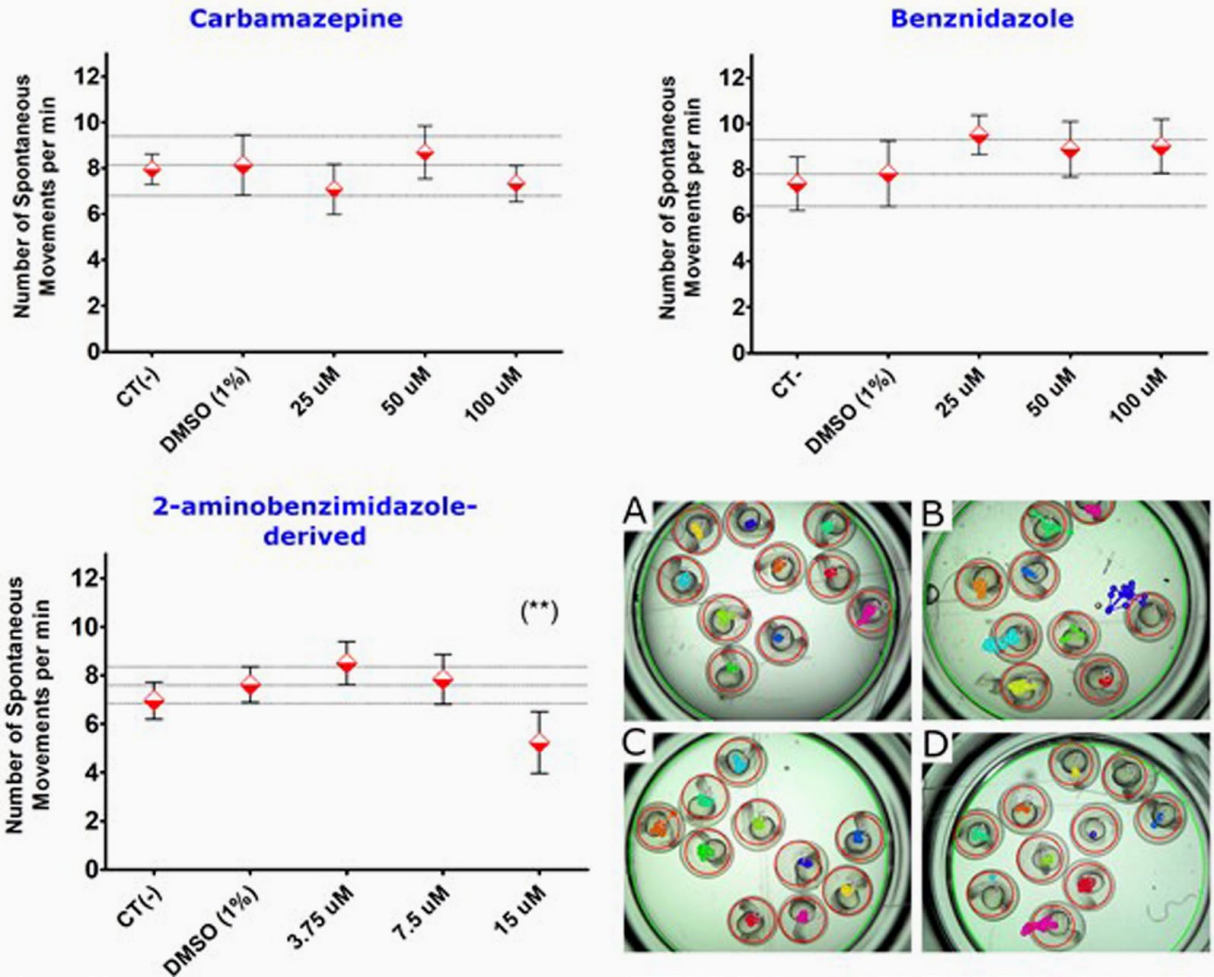
Plot showing the means values ± IC95 of the number of spontaneous movements per minute of *Danio rerio* embryos 24 hpf exposed to different concentration of carbamazepine, benznidazole and 2-aminobenzimidazole derived. Images of well showing the results of spontaneous movement analysis performed on embryos with 24 hpf, submitted to DMSO (A) or treated with the compounds benznidazole 100 µM (B), carbamazepine 100 µM (C), and 2amnbzl-d 15 µM (D). The colored dots represent the movements performed by each embryo. These results were generated by the SOFTWARE ESPMOV Count v1.0 that analyzes the number of spontaneous movements from each embryo. The data were analyzed using ANOVA statistical tests and Dunnett's Multiple Comparison Test. (**) *p* < 0.001

It was observed neurotoxic effects in groups of embryos exposed to 15 µM of 2amnbzl-d (Fig. 7). It was no differences between spontaneous movements of the embryos exposed to carbamazepine and benznidazole compared to the 1% DMSO group control, indicating the absence of a neurotoxic effect.

## Discussion

Zebrafish embryos develop most of the major organ systems present in mammals, including the cardiovascular, nervous and digestive systems, in in less than a week and most of the safety and efficacy assays can be carried out with zebrafish embryo and larvae with data generated biologically relevant [4]. To endorse the 3Rs principles, the tests chosen for the toxicological screening used zebrafish embryos and larvae up to 120 hpf, classified as in vitro models by international Directives.

2amnbzl-d compound showed important toxicological findings in mice and its drug discovery campaign was stopped due to toxicity, among other reasons. For the purpose of comparing the results obtained with a new chemical entity still going through early drug discovery stages, two approved drugs on the World Health Organization´s list of essential medicines, benznidazole and carbamazepine, were used since their toxicological profile is well known. Carbamazepine, a commonly prescribed agent for focal epilepsy, neuropathic pain, schizophrenia and bipolar disorder [14], common side effects include nausea and drowsiness, serious side effects may include skin rashes, decreased bone marrow function, suicidal thoughts, or confusion. and care should be taken in pregnancy in those with either kidney or liver problems [15]. Bradycardia has been describing in patients exposed to high dose of carbamazepine [16]. Benznidazole, a drug used for specific treatment of Chagas disease [17], common side effects include rash, numbness, fever, muscle pain, loss of appetite, and trouble sleeping. It is not recommended during pregnancy or in people with severe liver or kidney disease [18].

The zebrafish could be used to accurately predict relative acute toxicity as rat inhalation, rabbit dermal, and rat oral exposures through correlations to 96 hpf zebrafish LC50 values [6]. The acute toxicity of compounds was performed by OECD Directive 236 and the results demonstrated that 2amnbzl-d was potentially lethal (CL50% 14.8 µM) and teratogenic (EC50% 8.6 µM) when embryos were exposed to concentrations between 6.25 and 100 µM up to 96 hpf. The embryo developmental malformations observed, such as the absence of hatching and bradycardia, are directly correlated with prenatal loss in rabbits [6]. These results corroborate with a recent study carried out in rodents for the same compound Ferreira et al. [10], the authors observed mortality and distress in the mice that were submitted to efficacy tests, finally, the development of the compound was discontinued. Thus, the use of the zebrafish model could have predicted the acute toxicity of the compound within 4 days (96 h), and tests on rodents could be avoided. On the other hand, the compounds Carbamazepine and Benzimidazole did not cause changes in the development of the embryos when exposed to concentrations between 6.25 and 100 µM, which demonstrates that the OECD 236 test [10] predicted the safety of these compounds that are currently used in the treatment of human diseases and were previously approved in all preclinical and clinical trials.

An early in vitro safety assessment was conducted in which 2amnbzl-d showed low inhibition of the hERG channel (IC50 > 30 μM) and the main cytochrome P450 family enzymes (IC50 > 20 μM), similarly, screening against an off-target panel of a panel of 20 marks covering key human enzymes, kinases and receptors showed low interference in the activities (EC50 > 10 µM) as well [10]. On the other hand, 2amnbzl-d compound showed significant hepatotoxic effects at concentrations above 7.5 µM, as well as neurotoxic effect at concentration 15 µM in zebrafish. Zebrafish are vertebrates and therefore share a high degree of sequence and functional homology with mammals (more than 70%), including humans. Discordances between in vitro*/*in vivo assays are abundant. For example, in experiments using immortalized human liver epithelial (THLE) cells transfected with plasmid vectors that encoded human cytochrome P450s 1A2, 2C9, 2C19, 2D6, or 3A4a, for studied the behavior of 103 drugs, the authors found that the EC50 are far higher than the estimated C_max,u_ concentrations of the majority of the drugs in human peripheral blood. The authors justify the results, inferring possible suppression of P450 enzyme activity by DMSO, by difference in the accumulation of metabolites within hepatocytes in vivo at concentrations that are much higher than those in peripheral blood due to transport across or due to first pass metabolism [19]. Often, experiments of cytotoxicity are carried out using the MTT as indicator of cellular viability. In a study was showed that diverse compound such as drugs, nanoparticles and polypeptides interfere with MTT reduction rate under/overestimating of the cell viability [20]. Off-target activities of drug candidates observed during in vitro pharmacological profiling frequently do not translate to adverse events in human. Consequences of an off-target activity observed during profiling of a drug (AMG 337) being evaluated for treatment of solid tumors is reported. Screen of 151 potential off-targets, did not guarantee that during the clinical trials, headache cause by cerebral vasorelaxation emerged as the dose-limiting adverse events in the first-in-human trial [21]. In a review about translatability of preclinical science to human applications, authors point out that part of problem is associated with low prediction of cultured cells or the potential drugs did not have good safety feature in animals for unknown reason. The authors suggest that a strategy to improve translational research would be the identification of candidate drugs by screening the compound libraries using three dimensional (3D) organoids for rapid drug screening and model organisms such as *Caenorhabditis elegans* and zebrafish in high-throughput assays [22].

Due to the conservation of cell biological, developmental and metabolic processes across all vertebrates, studies in fish can give great insight into human disease processes, to date all proteins studied have a similar function in fish and mammals [23]. Thus, the 2amnbzl-d toxicological effects demonstrated in zebrafish and in rodents show that the initial in vitro assays were not very predictive of the toxicity of the compound.

In study of Ferreira et al. [10], based on several in vitro studies and in vivo pharmacokinetic profiling, 2amnbzl-d progressed to a 5-day tolerability study in healthy mice. Oral doses of 25, 50 and 100 mg/kg/day were evaluated to identify dosing regimens for the follow-up in vivo proof-of-concept study. In this study, the group tested with 25 mg/kg/day of 2-aminobenzimidazole derived was well tolerated, however, in the group of 50 mg/kg/day, from the 3rd day of treatment the animals showed some clinical signs such as piloerection, change in behavior and visible irritability. No changes were observed in internal organs after euthanasia in day 6. Regarding the maximum dose, due to the reduction in body weight and serious toxic signs, including mortality, studies were terminated on day 3 or 4 of treatment. These studies in rodents could be refined and/or avoided, if the results obtained in the zebrafish model had been considered before their realization. Certain zebrafish endpoints showed correlations to rodent and rabbit toxicity (LC50%), such as spontaneous movement (neurotoxicity), could be assessed even earlier at 24 hpf. Thus, using zebrafish as a model for toxicity testing would decrease testing times and costs substantially, in comparison to rodent and rabbit testing with the requirement of testing at age 8–12 weeks (rats) or at least 12 weeks (rabbits) and then observing the animals for a minimum of 14 days [6].

Finally, as for the cardiotoxic effects, 2amnbzl-d did not show effects in 96 hpf larvae heartbeat, but carbamazepine induced bradycardia at a concentration of 100 µM. Despite being a drug approved by regulatory agencies, carbamazepine is contraindicated in patients with heart problems and may cause some adverse cardiac effects, such as heart condition disorder, arrhythmia, atrioventricular block with syncope, bradycardia, congestive heart failure, worsening of coronary disease [24]. Heart abnormalities were also recorded in rat embryos when exposed to carbamazepine above 211.6 µM [25] and overdose were seem with dosages higher than 169.28 µM (usual therapeutic levels are 16.9 µM to 50.7 µM) [26–29]. On the other hand, there was no cardiotoxic effect of benznidazole in zebrafish larvae, just as there is no mention of heart problems caused by this medication in the package insert and in the scientific literature.

Due to their importance in the treatment of diseases, carbamazepine and benznidazole are on the World Health Organization´s list of essential medicines, but side effects are fairly common, which were also not detected in the Pltf-AcHpNrCd. Other studies in zebrafish evaluated exposure time or concentrations higher than those used in the present study and observed chronic effects in zebrafish early life stage test at 105 µM carbamazepine treated for 10 days [30] and found carbamazepine growth retardation above 129.5 µM and EC50% 366.05 µM [31]. Similarly to our study, Buchanan-Kilbey et al. [32], show that both in the control group and in those treated with benznidazole at concentrations of 3.7–300 µM, there was no significant phenotypic change in the embryos, such as (i) cardiovascular malformations, including any pericardial edema, changes in heart beat and in blood circulation, (ii) swimming behavior, as assessed by observing spontaneous events and responses evoked by a light touch on the embryo's head and finally (iii) embryonic death. Thus, the proposed platform was sensible to perform an effective screening for approval of new compounds, however, it did not predict the side effects of the drugs. New trials must be carried out to try to predict these side effects.

In the pharmaceutical industry, the majority of compounds subjected to pre-clinical tests fail, and this results in a loss of $ 294 million per drug, being that the cardiac, hepatic and neurological toxicities the most impactful [33]. According to international ethical regulations, zebrafish larvae up to 5 days post fertilization (dpf) are considered in vitro models and are accepted as an alternative to animal testing [5]. In view of the urgent demand for new drugs and the potential that zebrafish must accelerate this process by prioritizing safe compounds, as well as the need for higher ethical standards of toxicological screenings, the zebrafish model must be included in tests before study in mammals to attempt the 3Rs principle.

## Conclusions

As significant changes were found in the toxicity tests, the 2amnbzl-d is likely to cause acute toxic effects related to death or malformations, hepatotoxic and neurotoxic effects in concentrations above 4 µM. The present study demonstrated that the toxicity of 2amnbzl-d compound could have been predicted by the zebrafish model in just 5 days of evaluation and refined the use of mammals in subsequent tests.

## Methods

The project was approved by animal ethics committee from the Federal University of São Carlos (Number: 5085180319). Test compound 2-aminobendazole (purity > 95%), compound 29 [10] and the reference standards of two comparator drugs (benznidazole and carbamazepine) (purity > 99%) were kindly provided by the non-profit R&D organization Drugs for Neglected Diseases *initiative* (DND*i*) Latin America.

Toxicological profile of 2-aminobendazole were evaluated according to major endpoints requirement of the Food and Drug Administration (FDA), such as acute toxicity, cardiotoxicity, neurotoxicity and hepatotoxicity. As control, two FDA-approved compounds were screened for the same endpoints, Carbamazepine and Benznidazole. All tests were carried out blindly.

Zebrafish embryos and larvae used in the test were supplied by the Central Vivarium of the Federal University of São Carlos, SP, Brazil, whose matrices are kept in automated rack at 28° C, pH 7.0, in a photoperiod 14 h light: 10 h dark and are fed by the feed Gemma®, twice a day. To obtain the embryos, breeders were kept in the same aquarium overnight in the ratio of 2 males to 1 female and the embryos collected in the morning, 10 min after fertilization (mpf). Before testing, fertilized embryos were selected by observing cell divisions under a microscope (60mpf). The tests were conducted only when the fertilization rate was above 90%.

*(a) Embryotoxicity:* To assess acute toxicity, OECD guideline 236 was adopted, in which 200 individuals were screened for each compound. Embryos with 1.5 h post fertilization (hpf) were exposed to five logarithmic concentrations of compound for 96 h (n = 25) in individualized 96-well plates. The viability of the embryos was assessed daily according to the observation of endpoints lethality indicators such as: embryo coagulation, absence of detachment of the yolk sac tail, changes in the formation of somites and absence of heartbeat [7]. Changes in the development of the embryos were recorded daily for evaluation of teratogeny. All tests were performed with negative control group (E3, n = 25); plate control (E3, n = 4), positive control group (3.4 dichloroaniline—4 mg L^−1^, n = 25) and solvent control group (1% DMSO, n = 25).

*(b) Cardiotoxicity:* To evaluate the effect of the compounds on the heart physiology, the heart rate normalized, and non-linear variability parameters such as area of the of the *Poincaré Plot* and sample entropy were analyzed in 50 individuals that were screened for each compound following Cornet et al. [5]. The analyzed parameters were chosen based on its capacity in to evaluate the velocity and the rhythm of the cardiac contraction, often altered in case of cardiotoxicity effects [34].

After 4 h of drug incubation (96–100 hpf), zebrafish larvae were anesthetized by immersion in 0.7 mM tricaine methanesulfonate (A4050, Sigma-Aldrich, Saint Louis, MO, USA) diluted in E3 solution. Embryos treated with 10 µM of haloperidol were used as positive cardiotoxic control. The 1% DMSO treated embryos were used as negative cardiotoxic control. Individual hearts were recorded during 120 s each (Fig. 3). Videos were acquired with a high-speed recording camera (Hamamatsu C11440 ORCA-flash 2.8) at 50 fps and analyzed with the CardioCount 2 software (Lab. of Applied Immunology—UFSCAR, São Carlos, Brazil) to assess cardiac parameters. Three base 2 logarithmic dilution of each compound and control groups (n = 10) were performed.

*(c) Hepatotoxicity:* To determine the hepatotoxic effect, the liver staining methodology of Zebrafish larvae with Oil Red O [8] was used to screened 150 individuals. Oil Red O is a lysochrome dye used for staining triglycerides and neutral lipids. Two phenotypic outcomes related to abnormal liver function were analyzed to assess hepatotoxicity: hepatic steatosis and lipid retention in the yolk sac. After 72 hpf zebrafish larvae were exposed to each compound for 48 h and later stained to assess possible changes in liver metabolism. Three base 2 logarithmic dilutions of each compound and control groups (n = 25) were performed. Ethanol 2% was used as positive group control. The 1% DMSO was the negative group control. Subsequently, the larvae were fixed in 4% paraformaldehyde, washed in PBS and subjected to a discoloration process, with a solution of H_2_O_2_ (3%) and KOH (0.5%). After discoloration, the larvae were washed and evaluated under an optical microscope (Fig. 5).

*(d) Neurotoxicity:* To evaluate the neurotoxic effect of the spontaneous movement at 24 hpf was evaluated according to Menelaou et al. [35] to screened 150 individuals. 10mpf embryos were exposed for 24 h to three base 2 logarithmic dilutions of each compound (n = 30) and negative group control (DMSO 1%). The footage of embryo spontaneous movement was captured for 6 min, with a digital camera attached to an optical microscope. The counting of the movements of each embryo was performed using the EspMovCont software (Lab. of Applied Immunology—UFSCAR, São Carlos, Brazil), from the second minute of filming, totaling 5 min of counting (Fig. 7).

*(e) Compounds and concentrations:* The test concentrations were based on the maximum dilution of the compounds in E3 medium. Maximum concentrations that did not observe precipitation after 24 h were selected*.*

1. Carbamazepine: Molecular weight: 236.3; Solubility: 100 µM in DMSO (1%) in embryonic medium; Embryotoxicity: 6.25 µM, 12.5 µM, 25 µM, 50 µM, 100 µM; Cardiotoxicity, hepatotoxicity and neurotoxicity: 25 µM, 50 µM, 100 µM. Total number of individuals screened: 550.
2. Benznidazole: Molecular weight: 260.2; Solubility: 100 µM in DMSO (1%) in embryonic medium; Embryotoxicity: 6.25 µM, 12.5 µM, 25 µM, 50 µM, 100 µM; Cardiotoxicity, hepatotoxicity and neurotoxicity: 25 µM, 50 µM, 100 µM. Total number of individuals screened: 550.
3. 2-aminobenzimidalole derived (2amnbzl-d): Molecular weight: 336.4; Solubility: 50 µM in DMSO (1%) in embryonic medium. Embryotoxicity: 6.25 µM, 12.5 µM, 25 µM, 50 µM, 100 µM, Cardiotoxicity, hepatotoxicity, and neurotoxicity: 12.5 µM, 25 µM, 50 µM. Total number of individuals screened: 550.

### Statistical analysis

The comparison between the variables continuous of groups with normal distribution and equality of variances were analyzed using ANOVA statistical test (one way) and the Dunnett test for the post-hoc assessment. Otherwise, the Kruskal–Wallis and Dunn´s test were employed. The difference between survival curves were analyzed by Log-rank (Mantel-Cox) Test with *p* values corrected by Bonferroni´s methodology. The software used was the GraphPad Prism 5 (GraphPad Software, San Diego, CA, USA). The comparison between the categorical variables of two groups were carried out using the Z score (https://www.socscistatistics.com) with *p* corrected using the Bonferroni´s methodology. The dose–response curves were plotted and analyzed by Probit Regression using the MedCal 19.8 software (MedCalc Software Ltd, Ostend, Belgium). The *p* values < 0.05 was considered significant.

## Data Availability

See “Methods” section.

## Abbreviations

2amnbzl-d: 2-Aminobenzimidazoles-derived
DNDi: Drugs for Neglected Diseases Initiative
EC50: Median effective concentration
FDA: Food and Drug Administration
Hpf: Hours post-fertilization
IC50: Median inhibitory concentration
LC50: Median Lethal Concentration
OECD: Organisation for Economic Co-operation and Development
